# Developing Smoke-Free Policies in Public Housing: Perspectives From Early Adopters in 2 Southern States

**DOI:** 10.5888/pcd15.170427

**Published:** 2018-06-21

**Authors:** Michelle C. Kegler, Erin Lebow-Skelley, Jaimie Lea, Adrienne M. Lefevre, Pam Diggs, Sally Herndon, Regine Haardörfer

**Affiliations:** 1 Department of Behavioral Sciences and Health Education, Emory Prevention Research Center, Rollins School of Public Health, Emory University, Atlanta, Georgia; 2 Youth Empowered Solutions, Raleigh, North Carolina; 3 Tobacco Prevention and Control Branch, Division of Public Health, North Carolina Department of Health and Human Services, Raleigh, North Carolina

## Abstract

**Purpose and Objectives:**

In 2016, the US Department of Housing and Urban Development (HUD) issued a new rule requiring smoke-free policies in conventional public housing by July 2018 (HUD, 2016). This process evaluation describes the policy development experiences of conventional and nonconventional public housing authorities (PHAs) in North Carolina and Georgia that had established smoke-free policies before the HUD rule.

**Intervention Approach:**

HUD began to issue guidance that encouraged smoke-free policies in public housing in 2009, and most early adopters were outside of the Southeast. Documenting the process early adopters in the Southeast used to develop their policies provides useful lessons for conventional PHAs and those with properties not covered by the rule.

**Evaluation Methods:**

Semi-structured interviews were conducted with PHA representatives from 23 PHAs with some level of smoking restriction, along with residents from 14 of these PHAs, from January to August 2016.

**Results:**

Organizational leaders and board members were usually the primary players in making the decision to adopt a policy, with approval processes consistent with any type of policy adoption. Common reasons for establishing the policy included costs of turning a unit; health of children, nonsmokers, and staff; HUD guidance; and concerns or experience with fire caused by cigarettes. Levels of restriction were influenced by layout of the property, perceptions of compliance and enforcement challenges, concerns about smokers congregating, resident mobility, weather concerns, consistency with HUD guidance, and availability of funds for designated smoking areas. Resident input was obtained through general meetings, resident advisory boards or councils, surveys, and formal comment periods.

**Implications for Public Health:**

Understanding the process of policy development and adoption enables public health practitioners to be more effective partners in advising on the flexible components of the HUD smoke-free rule and accelerating the adoption of comprehensive policies within nonconventional PHAs.

## Purpose and Objectives

In late 2016, the US Department of Housing and Urban Development (HUD) issued a new rule requiring smoke-free buildings and a 25-foot buffer zone in conventional public housing by July 2018 (1). The rule is designed to protect the estimated 2.1 million residents who live in public housing in the United States from secondhand smoke (SHS) (1,2). Although major dimensions of the policy are mandated, flexibility remains in whether areas outside of the buffer zone will be smoke-free (eg, common areas, parking lots, or all grounds), whether designated smoking areas will be established, and whether electronic nicotine delivery systems (eg, e-cigarettes) will be included (3). Public housing authorities (PHAs) also have flexibility in their enforcement strategies (eg, number of warnings, lease termination proceedings).

Smoke-free policies in multi-unit housing (MUH) are essential because voluntary smoke-free policies in units are not able to fully protect children and nonsmokers from SHS. SHS can travel between units and expose nonsmokers to SHS even if they do not allow smoking in their own living space (4,5). One nationally representative survey of US adults who lived in MUH and reported no smoking in their home during the past 3 months found that 16% reported smoke incursion into their units, and 29.5% reported incursion in their buildings (6). In the same study, respondents who lived in government-subsidized housing were more likely than MUH residents in unsubsidized housing to report smoke incursion into their unit.

Studies of smoke-free policies in MUH have largely focused on reasons to go smoke-free from the perspective of decision makers and residents. Most of the studies are survey-based and summarize resident, owner, and property manager perceptions of costs and benefits associated with smoke-free policies, as well as factors associated with positive opinions or existence of smoke-free policies (7–12). None have systematically evaluated the policy-making process, especially from the perspective of multiple PHAs.

We describe the policy development experiences of PHAs in 2 politically conservative states, North Carolina and Georgia, who established smoke-free policies before the HUD rule. The policy-making process is typically conceptualized as having 4 stages: formulation, enactment, implementation, and maintenance (13,14). Formulation includes prioritizing the problem, reviewing evidence, selecting approaches, and drafting policy solutions (14). Enactment includes engaging policy makers and champions and enacting the policy. Our evaluation objectives were to understand the steps taken in developing smoke-free policy, with an emphasis on the formulation and enactment stages, often referred to as development and adoption within the organizational policy context. Understanding these issues will aid public health practitioners to advise on the flexible components of the HUD rule in conventional public housing and facilitate policy adoption in subsidized housing not covered by the new HUD rule.

## Intervention Approach

Smoke-free policies that restrict smoking inside buildings reduce SHS exposure, reduce fire risk, and save costs from reduced health care use, renovation, fire, and productivity losses (15–20). Before the rule, HUD had strongly encouraged PHAs to establish smoke-free policies, and 678 had done so, generally in the Northeast, West, and Northwest (3). Many toolkits and support materials were developed to assist in the process, and state health departments have actively supported PHAs in developing smoke-free policies and provided cessation assistance to residents (21,22). Although retrospective in design, this process evaluation documents the policy-making process among the early adopters of smoke-free policies in North Carolina and Georgia.

## Evaluation Methods

### Study participants

We sought to conduct semi-structured interviews with at least 20 PHAs in North Carolina and Georgia that had adopted smoking restrictions for at least one of their properties. We began by sending targeted recruitment messages to the 17 PHAs in these 2 states that HUD reported as having smoke-free policies. There are 185 PHAs in Georgia and 98 in North Carolina who manage low-income MUH, but most were not listed as having smoke-free policies at the time of the study. We identified additional eligible PHAs through existing collaborations, state website listings of smoke-free housing, association listservs, and snowball sampling. We interviewed all the identified eligible PHAs in North Carolina and all but 6 in Georgia, with 13 interviews conducted in North Carolina and 10 in Georgia (n = 23). Of the 6 PHAs in Georgia who did not agree to an interview, 3 said they did not have time, and we did not reach the other 3 by the end of our call protocol. We asked to interview the person who was most knowledgeable about the smoke-free policy implementation process. At 3 of the PHAs this resulted in 2 people participating, resulting in 26 participants at 23 PHAs. After completing 23 interviews we had reached saturation with no new information obtained. Interviews were conducted from January 2016 to August 2016, and all but one were conducted in person.

We also interviewed 16 residents who lived at properties at 14 of these PHAs (ie, 2 group interviews). Residents were identified by the PHA representative we interviewed as involved in the smoke-free policy-making process or who lived at a property with smoking restrictions. We gave preference to residents who were active in a resident council or resident advisory board. Most of these interviews were conducted in person; 5 were conducted by telephone. The study protocol was approved by the Emory University Institutional Review Board and included signed or verbal consent from all participants and participant incentives of a $25 gift card.

### Interview guide

The full interview guide covered: decision-making process related to adopting a smoke-free policy and deciding the specifics of the policy, initial implementation, resident involvement, enforcement, and organizational and community context. We pretested the guide through key informant interviews with 3 PHA representatives not eligible for the study but knowledgeable about smoke-free policies. Interviews averaged 60 to 90 minutes for PHA representatives and 30 to 45 minutes for residents.

### Data analysis

All interviews were audio-recorded and transcribed verbatim. The initial codebook was based on the interview guide and an earlier pilot study on smoke-free policies in market rate housing; additional codes were identified through open coding of the first few transcripts. The full analysis team coded the first several transcripts together to refine the codebook and remove ambiguity in the code definitions. Once all team members were coding consistently, the remaining transcripts were double coded with discrepancies resolved through discussion. NVivo 10 (QSR International) was used for data management and analysis. After all transcripts were double coded, reports were generated for specific codes (eg, champions), with a second round of inductive coding to identify themes in that code (23). Tentative themes were placed in matrices, with themes as rows and descriptors as columns (ie, conventional vs nonconventional; relationship with health department). Cells were populated with transcript identification to serve as an audit trail to enhance trustworthiness of the findings and to identify patterns by characteristics of the PHA (24).

## Results

### Description of PHA representatives and PHA residents

Eight PHAs were conventional in that they directly owned and managed properties funded through the Public Housing Program of HUD. Fifteen were nonconventional, having funding streams such as the Housing Voucher program in addition to or exclusive of the Public Housing program. A PHA was also considered nonconventional if it outsourced management to a privately owned management company. Most PHA representatives we interviewed were directors or chief executive officers (n = 11), directors of asset or property management (n = 5), or chief operating officers (n = 3). Most were female (n = 17), aged 36 to 50 years (n = 13), and white (n = 18). Most had a college education (n = 24) and were nonsmokers (n = 24) (Table 1).

**Table 1:** Characteristics of Representatives and Residents Interviewed to Assess Development Process of Adopting Smoke-Free Policies, 23 PHAs in North Carolina and Georgia, 2016

Characteristic	PHA Representative (n = 26)^a^	Resident (n = 16)^b^
**Type of PHA**		
Conventional	9	6
Nonconventional	17	10
**Years with PHA**		
<1	1	NA
1–2	3	
3–5	2	
6–10	11	
≥10	9	
**Title**		
CEO or Director	11	NA
Chief Operating Officer	3	
Director of Asset/Property Management	5	
Other	7	
**Years lived on property**		
<1	NA	1
1–2		3
3–5		2
6–10		2
>10		8
**Resident council member**		
Yes	NA	6
No		10
**Sex**		
Male	9	10
Female	17	6
**Age, y**		
18–35	4	1
36–50	13	6
51–65	9	5
≥65	0	4
**Race/ethnicity**		
White	18	2
African American	7	11
Hispanic	1	0
More than one race/mixed	0	2
Other	0	1
**Education**		
Grades 9–12	0	7
High school graduate/GED	0	2
Some college/trade school/associates degree	2	6
College graduate	18	0
Graduate degree	6	1
**Smoking status**		
Nonsmoker	19	8
Former smoker	5	2
Current smoker	2	6
**E-Cigarette use**		
Never tried it	23	11
Tried it	2	5
Current user	1	0

Abbreviations: GED, general educational diploma certificate; NA, not applicable; PHA, public housing authority.

a Representing 23 PHAs: 8 conventional and 15 nonconventional.

b Representing 14 PHAs: 5 conventional, 9 nonconventional.

Table 1 also presents demographic and other relevant characteristics of the resident participants. Six of the 16 were members of a resident council, and 8 had lived at the property for more than a decade. Most were male (n = 10) and African American (n = 11) with less than a high school education (n = 7) or some college/trade school (n = 6). Six were current smokers, and most had not tried e-cigarettes (n = 11).

### Description of the PHA smoke-free policy

Most PHAs had a smoke-free policy that banned smoking inside buildings (ie, residential units and administrative offices). Two had comprehensive policies that banned smoking property-wide including outdoors (Table 2). Common policies included allowing smoking anywhere outdoors, restricting smoking to designated outdoor areas, and buffer zones of various widths. At least 16 of these policies, including 7 from conventional PHAs, did not conform to the new HUD guidelines. Only 5 PHAs adopted smoke-free policies before 2011 (Table 2).

**Table 2:** Description of Policy, Study on Development Process of Adopting Smoke-Free Policies in 23 PHAs in North Carolina and Georgia, 2016

Policy Description^a^	No.
**Overall policy**	
Comprehensive (indoors and outdoors)	2
All indoor spaces smoke-free	20
Common areas only	1
**Outdoor policy specifics^a^**	
Buffer zones of 10, 15, 20, or 25 feet	7
Designated areas only	6
Back porches only	2
Anywhere but common areas	1
Anywhere outdoors	6
**Year of policy adoption**	
2009–2010	5
2011–2013	10
2014–2016	8

Abbreviation: PHA, public housing authority.

a Some participants described varying policies across properties.

### Policy formulation or development

**Players involved in the decision-making process.** We asked participants to identify who had been involved in the decision-making process for adopting a smoke-free policy. Board members and organizational leaders (eg, executive director) were mentioned most often. More than half had involved residents in the decision-making process, with more than one-third formally involving their resident advisory council or board in the decision.

**Reasons for initiating a smoke-free policy.** The most commonly cited reasons for adopting a smoke-free policy were the costs of turning a unit, health of residents and staff, HUD guidance and concerns, or direct experience with fire caused by cigarettes (Table 3). Additional reasons included an organizational culture that emphasizes health or doing “what’s right,” public norms for smoke-free environments, board encouragement, a desire to keep new and renovated buildings clean, resident demand, and external influences such as health department support or LEED (Leadership in Energy and Environmental Design) certification.

**Table 3:** Most Common Reasons for Initiating a Smoke-Free Policy, Study on Development Process of Adopting Smoke-Free Policies, 23 PHAs in North Carolina and Georgia, 2016

Theme	Illustrative Quote
**Cost of turning a unit**	. . . [B]y the time we get in there it’s brown. And we’ve had to replace all the drywall and everything because putting KILZ on it, the tobacco seeps straight back through. So it’s insane. . . . I mean, if you were to just paint it without putting the KILZ on the paint . . . the labor for that is $600. So to put KILZ on you can pretty much double [the cost of the] paint job. And if you have to take down drywall you can pretty much triple it. Not only that, but if you have to take out the tile, you never know. (Nonconventional PHA representative, Georgia)
**Health concerns for residents and staff**	When I have maintenance people that go in these apartments and work 8 hours a day, you know, in and out, and sometimes a job might take 30 minutes or an hour inside an apartment, so these guys, in our case, we have 2 guys . . . walk in the apartments, and they do their job for 8 hours a day, they go home smelling like they've smoked a pack of cigarettes, and their lungs probably feel like they smoked a pack of cigarettes. Neither one of them smoke, their families don't smoke, and I don't smoke. None of my staff smokes, but when I would go into an apartment just to have something signed, I'd smell like smoke the rest of the day. And so, you know, secondhand smoke, I've always read and heard that it's even worse on a person than a smoker, because your lungs are not used to all that tar and nicotine and it's not already corroded, so they absorb a lot quicker, your lungs do. And so, I mean, I just didn't feel like it’s fair to ask our people to go inside these apartments and do work, if they didn't smoke and didn't want to be around smoking, but yet their job tells them you got to, you know? (Nonconventional PHA representative, Georgia)
**HUD guidance**	We had already had some discussions, you know, about the smoke free and then HUD came out with recommending that housing authorities move in that direction. We took some time. You know, like I said, it took a few years to make a decision to go ahead, so probably had HUD not recommended it, it may not have come as soon as it did, you know? (Conventional PHA representative, North Carolina)
**Concerns about fire**	We've had several issues with smokers who fell asleep, caught stuff on fire. It's a huge safety thing. Health-wise, I think it's smart to not do it. If you want to smoke, you can go elsewhere. You don't need to be around other people, and safety-wise, take it out in the open air over, you know, over across the parking lot, because I don't want you falling asleep and hurting anybody else. I think it's a smart thing. I think in the long run it will help save money and save lives, and I think it's, it's about time that HUD rules caught up with the direction stuff is going. I mean, restaurants implemented it years ago, so this is — this is just keeping up with the times and the whole effort to be healthy. So I think it's a good thing, and I think it's pretty neat that we were ahead of that and already converting our properties to a nonsmoking property. (Nonconventional PHA representative, Georgia)

Abbreviations: HUD, US Department of Housing and Urban Development; PHA, public housing authority.

**Resources used in planning the policy.** PHA representatives discussed the resources they used in developing their policies. HUD guidance and toolkits were mentioned most often, followed by legal counsel and internet searches. Health departments were also mentioned, with an emphasis on cessation support and useful resources such as sample resident surveys, policy language, and presentations on the rationale for and legality of smoke-free policies.

**Decisions about level of smoking restriction.** Respondents provided significant detail about the thought process and steps in making decisions about policy specifics, such as whether the policy should be property-wide or only in common areas and living units, and whether buffer zones or designated smoking areas should be established. One of the first decisions was whether to go smoke free across the property including outdoors. PHAs with comprehensive policies felt that enforcement would be easier with no smoking allowed anywhere and that comprehensive policies were consistent with policies implemented elsewhere such as hospitals and other public places. Concerns about smokers congregating, monopolizing common areas, and littering the grounds with cigarette butts were also mentioned as reasons to go smoke-free property-wide. Additionally, PHAs with comprehensive policies had at least some new or rehabilitated buildings.

Those not establishing smoke-free properties were concerned about forcing residents on to the street or other properties, resident mobility, bad weather, and consistency with HUD guidance (ie, 25-foot buffer). As one participant described:

We looked at the layout of the grounds, and we tried as much as possible to go almost entirely as smoke free on grounds. The challenge that we had in several of our properties was just locational issues. So we’re in an uptown environment, which means if we push them off our property, they're on the city sidewalks, and then we’re being a bad partner to our other businesses and partners in the city, or we in one case are bordered by county parks, and county parks have a ban from public smoking. (Nonconventional PHA representative, North Carolina)

Another decision was whether to have a buffer zone, a designated smoking area, or both. Those selecting a buffer zone with no designated smoking area mentioned cost of creating a designated area, not wanting to create a gathering spot for smokers, concern over nonsmokers who live near the designated area, and easier compliance. Having an area that could easily serve as the designated smoking area facilitated selection of a designated area. A few chose to allow smoking near the building, citing concerns about resident mobility, adverse weather, encouraging compliance, easier enforcement, and lack of funds to erect structures.

**Inclusion of e-cigarettes in prohibited products.** E-cigarettes were included in fewer than half of the policies. When included, fire risk and lack of safety evidence were key reasons. When not included, the most common reason was that the policies were passed before e-cigarettes were popular.

### Adoption of the smoke-free policy

**Typical approval process.** Typically, staff drafted the policy and then obtained resident input, often through meetings with resident councils. The policy and resident comments were then presented to the board for approval. A few representatives from conventional PHAs described a formal process with a 30-day comment period. The need for HUD approval was mentioned by a few nonconventional PHAs. More conventional PHAs than nonconventional PHAs described an approval process that involved residents.

**Obtaining resident input.** Almost all of the conventional PHAs reported they obtained resident input at some point during the process, as did most nonconventional PHAs. Input was most commonly obtained before the decision or to inform policy details. Methods included general meetings with residents, resident advisory boards or councils, surveys, or formal comment periods. Some of the meetings were described as heated, but most residents typically supported the policy:

We kind of drafted a policy, and then we had resident meetings and said, “we’re considering doing this, what do you think about it?” And generally we found that the residents who did not smoke thought it was a great idea; some of the residents who did smoke thought it was a good idea, too, because they were trying to quit; and then some residents who did smoke said we're violating their . . . rights, when they ought to be able to do what they want to do in their own apartment. So we had a mixture of that kind of information. We found, though, that there seemed to be more weight to . . . having folks not smoke than smoking and that we were still allowing them to smoke, they just could not do it inside the apartment. We weren’t telling them you couldn't smoke at all. (Conventional PHA representative, Georgia)

Existing structures for resident engagement were commonly used as part of the policy-making process, with resident advisory boards or councils often mentioned, as well as resident representation on boards of directors or boards of commissioners.

Now the housing authority of [name], on its board of commissioners, has a full resident commissioner representative that’s a voting member of the board. And that is that representative that’s going to help and be there to help move policy forward. And we also have 2 resident advisors that sit on the housing authority board of commissioners. They do not have voting rights, but they have the opportunity and they are asked to bring insight from the residents’ perspective regarding the impact of policy decisions. So that is how the housing authority ensures that the resident voice is heard at the policy-making level. (Nonconventional PHA representative, Georgia)

Some of the discussion of resident input centered on a HUD-required comment period for policy changes:

Well, they come to the hearings and they can express if they like it, they can ask questions. They can tell us if they do like it, what they don’t like, why it may not work. They have a chance to come out to that hearing and they have a chance to express what they like and don’t like at that time and we take all the notes and we write down every complaint, good or bad, we write them all down, compile them, and then they’re attached to our hearing so that we’ll have it. So when we present to the board, the board can see that 15 residents showed, 10 were against it, 4 or 5 nay, you know, abstained, so we keep those notes to compile it. (Conventional PHA representative, Georgia)

Even when input was not required by HUD, PHAs generally built it into their process:

Not really there, because of that property being different and it’s under a different part of HUD, all we had to do was post it for 30 days. We did have resident meetings though. We always do that anyway. We go over and above what really is required. So we had a couple of resident meetings. They weren’t necessarily pleasant meetings but then we posted it for 30 days and then went to our board for approval after that and then it was implemented. What we did is, we gave them 6 months warning. Said, okay, in 6 months, as you start signing your lease, each time you sign your renewal, you’ll also sign this addendum. And we have an addendum that we require. But every new person that came in, came in under that policy so they had to sign the addendum as they moved in. And we felt like giving them 6 months plus was fair warning. And then, yeah, we listed resources. Smoke cessation groups and that sort of thing. (Nonconventional PHA representative, Georgia)

Although only mentioned once or twice, building support for the policy in the broader community, particularly with legal aid and local magistrates, was seen as useful. This occurred after the policy was passed but before it was implemented:

So after our board approved it but before we hit the ground running with it, we actually re-met with our resident advisory board and legal aid to sort of explain this is where we've gone, and . . . our resident advisory board really stood behind us and said, “We want this, we're going this way, so help . . . with the enforcement here and kind of stay out of the way of legal aid,” so to speak. They said it much more politely than that, but that was the gist of it. So we met with them. We also met with our magistrates in the county and said, this is something we're doing, we want to talk about it, we want to know what you think, but we want you to know it's coming and we need your support. When we get somebody who violates the policy, we need to be able to uphold it, and so I think that was a good preemptive work before we got there, because we have not seen a lot of pushback from legal aid or . . . anyone to that matter related to our smoke-free policy. I won't say that related to anything, but related to the smoke-free policy. (Nonconventional PHA, North Carolina)

Surveys of residents, conducted by approximately half of the PHAs, were viewed as very helpful, mainly in showing that most residents supported a smoke-free policy. Newsletters and letters to residents and informal meetings were also mentioned. Input was used in a range of ways, most often to build support for the policy before adoption or implementation. Input was incorporated into policy or implementation details such as where to locate designated smoking areas. A few participants commented that resident participation in surveys or meetings was low. A few participants did not obtain resident input because of lack of mandatory structure to do so or that the policy would be implemented in new construction.

**Resident descriptions of their involvement.** Residents described their involvement, or lack thereof, in the policy adoption or implementation process at their PHA. Close to half of the residents we interviewed felt they were involved in some way, whether it was before or after the decision to adopt a policy had been made. Others were unable to recall the process and a few reported they did not know about the policy until it had been decided on, although one of those residents said that the PHA compromised on the implementation timeline after meeting with residents. Residents mentioned surveys, meetings, and resident advisory boards as methods of involvement. A resident association president described:

They sent me a letter. Well, they sent everybody a letter. Myself, to the treasurer, secretary, and assistant treasurer. They sent all 4 of us a letter. These are the 4 people that was there . . . not the whole. So they sent all of us a letter. Whenever they getting ready to do anything, us 4, we’ll get a letter . . . They going to do something, they want us involved, they send us a letter to let us know and then this way, we can bring it back to the association and discuss it with them and see what kind of feedback we get from them. (Nonconventional PHA, Resident Association President, Georgia)

**Champions and opponents.** Participants were asked if there were any champions who advocated for the smoke-free policy or if there was anyone who strongly opposed the policy. The most common champion for PHAs was the most senior person in charge (eg, Executive Director). Other champions for PHAs included board members or a staff member. Only a few participants said they did not have a champion for their policy. An executive director commented:

Well, I can’t — I won’t take credit for it because . . . it was my idea that we do it, but I did a lot of research on other people's policies. I also belong to some national – I said – I mentioned GAHRA, the state association, but not many people in GAHRA had done that, only a couple, and so I — I also sit on some committees with some national groups, and so I talked to a lot of people throughout the United States, and some of them have smoke policies. So I got a lot of other policies and looked at them. (Nonconventional PHA representative, Georgia)

Only 2 PHA representatives reported having an opponent to the policy. One PHA said the challenger was on staff. The other PHA reported the opponents were board members. One opponent was approached by engaging them in discussions on the benefits of and reasons for the policy and the other was satisfied when grandfathering of current smokers was included in the policy.

### Patterns in the policy development and adoption process, by PHA characteristic and relationship with health departments

Slightly fewer than half of the PHAs partnered with their health department during the policy development process. These PHAs were more likely to have cited HUD guidelines and health as an initiation reason, and slightly less likely to have cited cost or fire, compared with those who did not work with their health department. Fewer of the earliest adopters had worked with their health department, possibly because health departments were not working on this issue yet. More of the later adopters had partnered with their health department than not. Interestingly, more conventional PHAs worked with their health department during the policy development process than nonconventional PHAs. Most PHAs who adopted policies before 2011 (n = 5) had stricter policies (eg, comprehensive, designated area, buffer zone), were in urban areas of Georgia, did not partner with their health department, and cited cost as one reason they initiated the policy. Conventional PHAs were more likely to describe resident engagement in the policy development process.

## Discussion

We examined the policy development and adoption process undertaken by conventional and nonconventional PHAs before the new HUD rule. We found that most PHAs used a process consistent with published HUD guidance. Steps in the HUD-recommended process are: develop the policy; communicate the policy change to residents, staff, and board members; obtain board approval; obtain resident input; ensure residents sign lease or lease addendum that includes the policy language; update house rules; and implement and enforce the policy (3). In general, smoke-free policy development was seen as following the same process used for any policy or rule change covered by HUD regulations and requirements.

Now that a smoke-free policy will be mandatory for conventional public housing, resident and board input will likely focus on the flexible aspects of the smoke-free policy, including whether to include other tobacco products such as e-cigarettes and whether to extend smoking restrictions beyond the required 25-foot buffer (3). Our study identified a series of considerations in making these determinations, such as physical layout of the property, resident engagement and feedback on policy decisions such as designated smoking areas, feasibility of enforcement, and facilitating compliance. Our study also highlighted that many PHAs have properties not covered by the new HUD rule. Our findings on the process are relevant in these settings and suggest that the same process should be followed.

Most PHAs, both conventional and nonconventional, engaged residents to gain support for smoke-free policies and, in some cases, to help decide policy specifics around issues such as designated smoking areas. To engage residents, most PHAs used existing organizational structures, such as regular resident meetings, formal comment periods, and resident advisory boards or councils. Although resident input may not be mandated, in many cases it was viewed as consistent with practices and values of the PHAs, and resident surveys helped shore up support for the policy, as most residents were almost always in favor of the policy. This is consistent with resident surveys conducted elsewhere (9,25–28).

We found that PHAs in the Southeast shared similar reasons as multi-unit housing owners and managers in other areas of the country for adopting smoke-free policies (7,8,10,29). Emphasizing health benefits, cost savings, reduced fire risk, and nonsmoking norms as reasons for adopting smoke-free policies should continue to be important for expansion of such polices to other forms of subsidized housing. The impetus for adoption of smoke-free policies often came from a high-level champion who advocated for the policy. Although smoke-free policies are now required for conventional public housing, champions are still important for building support among staff and residents and to shepherd them through the modifications, such as increased size of buffer zones or elimination of grandfathering clauses, required to meet the new rule. Moreover, champions will still be critical for expansion of the policies to subsidized housing not covered by the new HUD rule.

We examined whether type of PHA or relationship with a health department influenced the policy development process. Compared with the earliest adopters of smoke-free policies in Georgia and North Carolina, many PHAs had recently partnered with health departments — perhaps because of the Community Transformation Grants program started in 2011, which included funding to promote the adoption of such policies. However, health departments can still work to reach more PHAs, especially by reaching out to nonconventional PHAs and using information about fire risk and cost savings to target PHAs who may not be as concerned with resident health. Reaching nonconventional PHAs is important since they have been less likely to partner with the health department and have properties not required to comply with the new HUD rule.

Our study has limitations. We generally interviewed only one representative per PHA, and in most cases several years had passed since the policy had been adopted, which may have affected the quality of representatives’ recollections of some of the details of the policy-making process. We also interviewed just one or 2 residents per PHA, and not all of them had been present when the policy was passed, thus diminishing the utility of these interviews for representing resident perspectives at the time of policy adoption. Lastly, social desirability bias may have been a factor, as respondents knew the interviewers were from a school of public health and, in North Carolina, also affiliated with the state health department. This awareness most likely affected responses related to the reasons for policy adoption (eg, health of residents and staff) rather than the adoption process itself.

Future implementation research could identify factors that influence implementation success. Implementation success should be defined and measured, perhaps by indicators of compliance or enforcement (eg, number of violations, number of evictions). Inclusion of a strong measure of implementation or enforcement success would help identify the most influential barriers and facilitators. Future research on smoke-free MUH policies could also examine the implications of including e-cigarettes and other alternative tobacco products in such policies and document the effects of comprehensive policies that cover the entire property including outdoor spaces versus those with only a 25-foot buffer zone on outcomes such as compliance or cessation.

## Implications for Public Health

Our study has implications for both public health professionals and PHAs, as the latter, especially those with properties not covered by the HUD rule, move forward in adopting comprehensive smoke-free policies. First, PHAs should treat smoke-free policies the same as they would any other policy and use the well-established processes already in place (eg, resident councils, resident advisory boards, 30-day comment periods) for input. Second, resident surveys, although not essential, are useful, particularly to document that most residents support a smoke-free property. Third, physical layouts (eg, garden-style buildings, shared hallways, distance to property boundaries) and available funding for creation of designated smoking areas should be considered when establishing policy specifics. Fourth, PHAs and their partners should build a case for smoke-free policies based on cost savings, reduced fire risk, and health benefits to persuade various stakeholders to support and accept the policy. Fifth, ample time for notification, along with support to smokers in encouraging them to quit, should be built into the process. Health departments can be valuable partners in helping to make the case for comprehensive smoke-free policies and for cessation resources. Sixth, smoke-free policies are easy to implement for all new buildings and all rehabilitated buildings. Seventh, grandfathering in current smokers can create confusion among new residents and enforcement challenges. Eighth, meeting with local legal aid offices and magistrates can help them understand the legality and benefits of smoke-free policies before any need for eviction court.

Our evaluation results identified decision points and processes that aid practitioners in better understanding how PHAs operate. This deeper understanding should be useful in providing support to conventional PHAs as they determine the details of their policy implementation process and to PHAs with mixed funding streams who are poised to be the next set of subsidized housing to experience a major shift toward smoke-free housing.
